# Diabetes in Old Male Offspring of Rat Dams Fed a Reduced Protein Diet

**DOI:** 10.1155/EDR.2001.139

**Published:** 2001

**Authors:** Clive J. Petry, Matthew W. Dorling, Dorota B. Pawlak, Susan E. Ozanne, C. Nicholas Hales

**Affiliations:** 1 Clinical Biochemistry Department University of Cambridge Addenbrooke's Hospital Hills Road Cambridge CB2 2QR UK; 2 Human Nutrition Unit Biochemistry Department University of Sydney NSW Sydney Australia

## Abstract

Restricted fetal growth is associated with increased risk for the future development of Type 2 diabetes in humans. The study aim was to assess the glucose tolerance of old (seventeen months) male rats, which were growth restricted in early life due to maternal protein restriction during gestation and lactation. Rat mothers were fed diets containing either 20% or 8% protein and all offspring weaned onto a standard rat diet. In old-age fasting plasma glucose concentrations were significantly higher in the low protein offspring: 8.4 (1.3)mmol/l v. 5.3 (1.3)mmol/l (p = 0.005), Areas under the curves were increased by 67% for glucose (p = 0.01) and 81% for insulin (p = 0.01) in these rats in intravenous glucose tolerance tests, suggesting (a degree of) insulin resistance. These results show that early growth retardation due to maternal protein restriction leads to the development of diabetes in old male rat offspring. The diabetes is predominantly associated with insulin resistance.


*Int. Jnl. Experimental Diab. Res.*, Vol. 2, pp. 139–143
Reprints available directly from the publisher
Photocopying permitted by license only

© 2001 OPA (Overseas Publishers Association) N.V.
Published by license under
the Harwood Academic Publishers imprint,
part of Gordon and Breach Publishing,
a member of the Taylor & Francis Group.
Printed in the U.S.A.

### Diabetes in Old Male Offspring of Rat Dams Fed a Reduced Protein Diet

CLIVE J. PETRY[a,*], MATTHEW W. DORLING[a], DOROTA B. PAWLAK[b], SUSAN E. OZANNE[a] and C. NICHOLAS HALES[a]

[a]*Clinical Biochemistry Department, University of Cambridge, Addenbrooke's Hospital, Hills Road, Cambridge, UK, CB2 2QR;* [b]*Human Nutrition Unit, Biochemistry Department, University of Sydney, Sydney, NSW, Australia*

*(Received 27 March 2001; In final form 20 May 2001)*

**Restricted fetal growth is associated with increased risk for the future development of Type 2 diabetes in humans. The study aim was to assess the glucose tolerance of old (seventeen months) male rats, which were growth restricted in early life due to maternal protein restriction during gestation and lactation. Rat mothers were fed diets containing either 20% or 8% protein and all offspring weaned onto a standard rat diet. In old-age fasting plasma glucose concentrations were significantly higher in the low protein offspring: 8.4 (1.3) mmol/l v. 5.3 (1.3) mmol/l ($p = 0.005$). Areas under the curves were increased by 67% for glucose ($p = 0.01$) and 81% for insulin ($p = 0.01$) in these rats in intravenous glucose tolerance tests, suggesting (a degree of) insulin resistance. These results show that early growth retardation due to maternal protein restriction leads to the development of diabetes in old male rat offspring. The diabetes is predominantly associated with insulin resistance.**

*Keywords*: Glucose tolerance; Hyperinsulinaemia; Intrauterine growth restriction; Low protein diet

### INTRODUCTION

Over twenty studies have now shown a link between indices of restricted fetal growth and the future development of Type 2 diabetes, the metabolic syndrome or insulin resistance (reviewed in[1]). In rats early growth restriction may be induced by feeding dams a reduced protein diet. Their adult offspring display a phenotype which shows some similarities to that of the metabolic syndrome in humans.[2,3] However despite the rat model having a greater age-related loss of glucose tolerance, frank diabetes has not been observed.[1,2] This may be because the rats have not been studied at an advanced age. We have therefore carried out studies of seventeen month old male rats to determine their intravenous glucose tolerance and glucose-stimulated insulin secretion.

### MATERIALS AND METHODS

### Animals

Female Wistar rats bred at the Dunn Nutrition Unit, Cambridge, who weighed 235–250 g, were mated and assumed to be pregnant when a vaginal plug was expelled. They were then fed *ad libitum* either a control diet (containing 20% (w/w) protein) or an isocaloric low protein diet (Tab. I) (from Hope Farms, Woerden, Netherlands) during gestation and whilst the pups were suckling. Two days after birth litter sizes were standardised to eight pups. At 21 days of age the offspring were weaned onto a standard rat diet (LAD1; from Special Diet Services, Witham, UK). Two male rats from each of five

*Corresponding author. Fax: + 44 1223 330598, e-mail: cjp1002@cam.ac.uk

TABLE I Composition of the control and low protein rat diets (g/100 g)

| | Control (20% (w/w) protein) | Low protein (8% (w/w) protein) |
|---|---|---|
| Mineral and vitamin mixture | 5.05 | 5.05 |
| Casein (88 g protein/100 g) | 22.0 | 9.0 |
| dl-Methionine | 0.22 | 0.08 |
| Maize Starch | 8.0 | 8.0 |
| Cellulose | 5.0 | 5.0 |
| Soyabean Oil | 4.3 | 4.3 |
| Cerelose (glucose) | 55.15 | 68.17 |
| Energy (MJ/100 g) | 1.54 | 1.53 |

litters for each group were randomly selected for the study. Only male rats were chosen since at fifteen months of age they had been the ones exhibiting hyperinsulinaemia.[2] In this study the rats (termed 'control' and 'low protein' rats according the diet that *their mothers* were fed) remained on the LAD1 diet for the remainder of the study. All animal procedures were performed under the Animals (Scientific Procedures) Act 1986.

### Surgical Procedures

Seventeen month old rats were anaesthetised with halothane (Fluothane; Zeneca, Macclesfield, UK) (4% in oxygen for inducing and 2% for maintaining anaesthesia). Sterile catheters (Esco Rubber, 0.5 mm bore, Bibby Sterilin Ltd., Stone, UK) were placed bilaterally into the jugular veins. The distal ends of the catheters were tunnelled subcutaneously and exteriorised at the nape of the neck. Each catheter was backfilled with heparinised saline (20 U/ml) prior to being plugged. To maintain patency, the catheters were flushed every day with saline. The animal was allowed to recover until it appeared to have 'normal' feeding, drinking and grooming behaviour (generally 2–3 days). The intravenous glucose tolerance test was only performed if the animal lost less than 5% of its initial body weight at any stage after the surgery.

### Intravenous Glucose Tolerance Test

Glucose tolerance tests were performed in fully conscious, unrestrained animals. Food was removed from their cages six hours prior to commencement of the test. 200 μl blood was collected into heparinised tubes for basal results. Then a dose of 1 g/kg body weight of glucose was infused (as a 50% (w/v) solution) into the dosing catheter over a period of 30 sec., followed by flushing with 500 μl saline. Subsequently 200 μl blood samples were taken and stored on ice 1, 2, 4, 6, 8, 10, 13, 18, 24, 30, 45, 60 and 90 min. after the glucose infusion. At 25 min. all the sampled blood was centrifuged, the plasma removed and frozen for analyses and the remaining red blood cells mixed and reinfused at 46 min.

### Laboratory Analyses

Plasma glucose was measured using the glucose oxidase method (Sigma Trinder kit, Sigma Chemical Co., Poole, Dorset, UK). Plasma insulin concentrations were measured using Linco Sensitive Rat Insulin radioimmunoassay kits and each measurement was performed in duplicate (Biogenesis Ltd., Poole, Dorset, UK).

### Statistical Analysis

Data are presented as mean (SD) except in the figure and comparisons between groups were assessed using the Mann Whitney U test. $P < 0.05$ was considered statistically significant.

### RESULTS

At two days of age the low protein rat offspring were significantly lighter than equivalent controls: 5.82 (0.27) g v. 6.52 (0.44) g ($p = 0.02$). This 11% reduction in body weights had reached 44% when the rats were weaned at 21 days of age: 29.63 (3.61) g v. 52.76 (4.01) g, respectively ($p = 0.004$).

Intravenous glucose tolerance tests were performed on the six rats in each of the two groups used (from five different litters in each group) which survived until they were approximately seventeen-months-old (Tab. II). At this age, after over sixteen months of the two groups being fed the same diet, the reduction in body weights of the low protein rats was approximately 14% prior to surgery, prior to fasting and immediately prior to the intravenous glucose tolerance test (all $p = 0.02$). Figure 1 shows the glucose and insulin curves from the glucose tolerance tests. Low protein offspring had significantly higher fasting plasma glucose concentrations (8.4 (1.3) mmol/l v. 5.3 (1.3) mmol/l; $p = 0.005$). They also had significantly higher peak plasma glucose concentrations (at 2 min.) (32.8 (6.3) mmol/l v. 20.1 (6.6) mmol/l; $p = 0.02$). The areas under the glucose curves were 67% higher in the low protein rats than in the controls (Tab. II; $p = 0.01$). Fasting plasma insulin concentrations were significantly higher in the low protein offspring (818 (649) pmol/l v. 361 (149) pmol/l; $p = 0.03$). The areas under the insulin curves were 81% higher in these rats ($p = 0.01$). Both phases of insulin secretion had significantly increased area under the curves ($p < 0.05$).

TABLE II Characteristics of the male rats used in this study and results from the intravenous glucose tolerance test. Data are mean (SD). NS = not significant, AUC = area under curve

| | Control | Low protein | p |
|---|---|---|---|
| n | 6 | 6 | |
| Age (months) | 16.9 (0.5) | 17.7 (0.7) | NS |
| Preoperative Weight (g) | 796.2 (72.8) | 687.5 (52.2) | 0.02 |
| Prefasting Weight (g) | 779.7 (71.2) | 677.7 (55.3) | 0.02 |
| Experimental Weight (g) | 770.4 (71.1) | 670.3 (53.0) | 0.02 |
| Plasma Glucose AUC (min.mmol/l) | 709.3 (195.0) | 1182.0 (197.6) | 0.01 |
| Plasma Insulin AUC (min.nmol/l) | 72.3 (22.9) | 131.2 (29.0) | 0.01 |
| First Phase Insulin (min.nmol/l) | 4.5 (2.5) | 7.2 (1.5) | 0.04 |
| Second Phase Insulin (min.nmol/l) | 67.8 (20.6) | 124.0 (27.7) | 0.006 |

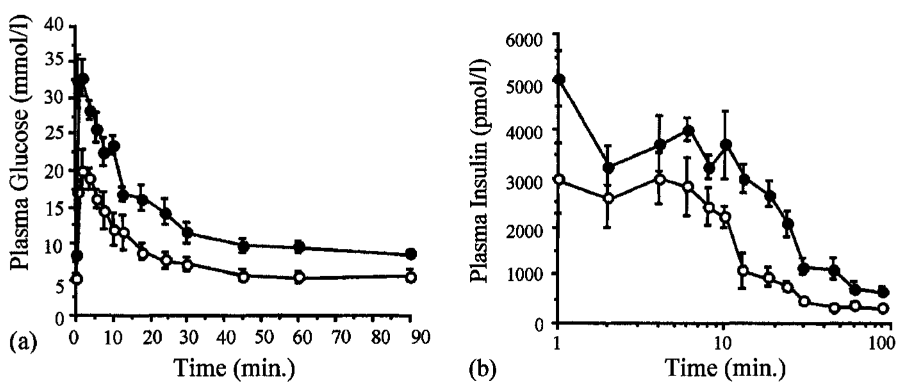

FIGURE 1 (a) Plasma glucose and (b) insulin concentrations after infusing 1 g/kg glucose into the jugular vein of seventeen-month-old male rats whose mothers had been fed a diet containing either 20% (w/w) protein ('control') or 8% (w/w) protein ('low protein') during pregnancy and lactation. n = 6 for both groups. Data are mean (S.E.M.). Key: ○ control rats, ● low protein rats.

### DISCUSSION

There is no formal definition of what constitutes diabetes in rats. However the presence of diabetes in the low protein offspring in this study is implied by the fact that their mean fasting plasma glucose concentration was higher than that considered to constitute diabetes in humans.[4] For the first time, therefore, maternal exposure to the low protein diet used in this study during gestation and whilst the pups were suckling has been shown to lead to both restricted early growth and the development of diabetes in male offspring in old age. This diabetes represents a worsening of glucose tolerance in comparison to the mild intolerance observed at fifteen months of age.[2] The mean age of death in these male rats (both controls and low protein offspring) is fifteen to sixteen months,[2] so the diabetes was observed in rats who had already lived slightly longer than average. Indeed four of the original control and four of the original low protein offspring had died by the time that the analyses were performed, of unknown causes. This suggests that previous attempts to observe frank diabetes in rat offspring of dams fed low protein diets were hindered by not allowing enough of the effects on glucose tolerance associated with the ageing process time to develop.[5]

The hyperglycaemia in these rats was associated with hyperinsulinaemia both after fasting and post-glucose load. This suggests that the diabetes was predominantly associated with insulin resistance. *In vitro* it has been shown that adipocytes from fifteen month old male low protein offspring have reduced insulin-stimulated glucose uptakes and reduced insulin-mediated inhibition of lipolysis.[6] This occurred when the animals had only a mild impairment of glucose tolerance and possibly, along with reduced insulin action in other tissues, may have contributed to the further deterioration in glucose tolerance observed in this study. The resultant compensatory hyperinsulinaemia in these rats was not sufficient to normalise the plasma glucose concentrations. This may have resulted from a slight impairment in the ability to secrete insulin, as was previously observed in younger rats whose mothers were protein restricted.[7] Both phases of insulin secretion were still enhanced in comparison to those of the controls, however. In humans, phase 2 but not phase 1 insulin secretion has been shown to be increased in men who were growth restricted during fetal life.[8]

The experimental diet used in this study was fed to the mothers of the rats with diabetes throughout the gestational and suckling periods. Recently discrepancies in blood pressure outcomes of similar dietary manipulations in rats have led to the suggestion that it is not the protein restriction *per se* that leads to disease but rather it is the interaction of this restriction with the other nutrients in the diet.[9] However these discrepancies were found using a low protein diet that was supplemented up to control levels with methionine and therefore may not reflect true protein restriction. Protein calorie restriction has previously been shown to lead to impaired glucose tolerance in young rats (reviewed in[5]), suggesting that it is indeed the protein deprivation that is critical for disease. At present it is not clear with this model whether it is the *in utero* exposure to the maternal protein restriction that is necessary for diabetes to develop or whether it is just the neonatal exposure or a combination of the two. At six weeks of age an actual improvement in the glucose tolerance of rat offspring was noted using the diet utilized in this study, independent of whether the exposure was before or after birth or a combination of the two.[10] In humans both restricted fetal growth and a combination of restricted fetal and neonatal growth have been shown to be linked to an increased risk of developing Type 2 diabetes.[5]

The thrifty phenotype hypothesis[5] suggested that the conflict between fetal malnutrition and subsequent adequate or over-nutrition coupled to ageing increases the risk of developing diabetes and the metabolic syndrome. This study shows that retarding growth by maternal protein restriction is a process which links restricted early growth to the future development of diabetes in rats and suggests that the mechanism linking these processes can occur independently of a genetic component. In humans such a process may also occur independently of a genetic mechanism, as suggested by studies in monozygotic twins who were discordant for diabetes.[11,12]

### Acknowledgements

We thank Professor I. D. Caterson at the University of Sydney for helpful discussions in setting up this study. The Medical Research Council and the Parthenon Trust provided financial support for the study. Expert technical assistance was provided by D. Hutt, A. Flack and A. Wayman.

### References

[1] Petry, C. J. and Hales, C. N. (1999). Intrauterine development and its relationship to type 2 diabetes mellitus. In: *Type 2 diabetes-prediction and prevention*, Edited by Hitman, G. A., pp. 153–168, Chichester, UK: Wiley.

[2] Hales, C. N., Desai, M., Ozanne, S. E. and Crowther, N. J. (1996). Fishing in the stream of diabetes: from measuring insulin to the control of fetal organogenesis. *Biochem. Soc. Trans.*, **24**, 341–350.

[3] Petry, C. J., Ozanne, S. E., Wang, C. L. and Hales, C. N. (1997). Early protein restriction and obesity independently induce hypertension in year old rats. *Clin. Sci.*, **93**, 147–152.

[4] Alberti, K. G. M. M. and Zimmet, P. Z. for the W.H.O. Consultation (1998). Definition, diagnosis and classification of diabetes mellitus and its complications. Part 1: diagnosis and classification of diabetes mellitus. Provisional report of a W.H.O. Consultation. *Diabet. Med.*, **15**, 539–553.

[5] Hales, C. N. and Barker, D. J. (1992). Type 2 (non-insulin-dependent) diabetes mellitus: the thrifty phenotype hypothesis. *Diabetologia*, **35**, 595–601.

[6] Ozanne, S. E., Dorling, M. W., Wang, C. L. and Nave, B. T. (2001). Impaired PI 3-kinase activation in adipocytes from early growth-restricted male rats. *Am. J. Physiol.*, **280**, E534–E539.

[7] Dahri, S., Reusens, B., Remacle, C. and Hoet, J. J. (1995). Nutritional influences on pancreatic development and potential links with non-insulin-dependent diabetes. *Proc. Nutr. Soc.*, **54**, 345–356.

[8] Flanagan, D. E., Moore, V. M., Godsland, I. F., Cockington, R. A., Robinson, J. S. and Phillips, D. I. (2000). Fetal growth and the physiological control of glucose tolerance in adults: a minimal model analysis. *Am. J. Physiol.*, **278**, E700–E706.

[9] Langley-Evans, S. C. (2000). Critical differences between two low protein diet protocols in the programming of hypertension in the rat. *Int. J. Food Sci. Nutr.*, **51**, 11–17.

[10] Shepherd, P. R., Crowther, N. J., Desai, M., Hales, C. N. and Ozanne, S. E. (1997). Altered adipocyte properties in the offspring of protein malnourished rats. *Brit. J. Nutr.*, **78**, 121–129.

[11] Poulsen, P., Vaag, A. A., Kyvik, K. O., Møller Jensen, D. and Beck-Nielsen, H. (1997). Low birth weight is associated with NIDDM in discordant monozygotic and dizygotic twin pairs. *Diabetologia*, **40**, 439–446.

[12] Bo, S., Cavallo-Perin, P., Scaglione, L., Ciccione, G. and Pagano, G. (2000). Low birthweight and metabolic abnormalities in twins with increased susceptibility to type 2 diabetes mellitus. *Diabet. Med.*, **17**, 365–370.

